# Birthplace in New South Wales, Australia: an analysis of perinatal outcomes using routinely collected data

**DOI:** 10.1186/1471-2393-14-206

**Published:** 2014-06-14

**Authors:** Caroline SE Homer, Charlene Thornton, Vanessa L Scarf, David A Ellwood, Jeremy JN Oats, Maralyn J Foureur, David Sibbritt, Helen L McLachlan, Della A Forster, Hannah G Dahlen

**Affiliations:** 1 Centre for Midwifery, Child and Family Health, Faculty of Health Level 7, University of Technology, 235-253 Jones St. Broadway, Sydney, New South Wales, Australia; 2 School of Medicine, University of Western Sydney, Sydney, Australia; 3 School of Medicine, Griffith University, Gold Coast, Queensland, Australia; 4 Consultative Council on Obstetric and Paediatric Mortality and Morbidity, Melbourne, Victoria, Australia; 5 Faculty of Health Sciences, La Trobe University, Melbourne, Australia; 6 Mother and Child Health Research Centre, La Trobe University and the Royal Women’s Hospital, Melbourne, Australia; 7 School of Nursing and Midwifery, University of Western Sydney, Sydney, Australia

**Keywords:** Birthplace, Perinatal, Maternity, Birth centre, Homebirth

## Abstract

**Background:**

The outcomes for women who give birth in hospital compared with at home are the subject of ongoing debate. We aimed to determine whether a retrospective linked data study using routinely collected data was a viable means to compare perinatal and maternal outcomes and interventions in labour by planned place of birth at the onset of labour in one Australian state.

**Methods:**

A population-based cohort study was undertaken using routinely collected linked data from the New South Wales Perinatal Data Collection, Admitted Patient Data Collection, Register of Congenital Conditions, Registry of Birth Deaths and Marriages and the Australian Bureau of Statistics. Eight years of data provided a sample size of 258,161 full-term women and their infants. The primary outcome was a composite outcome of neonatal mortality and morbidity as used in the *Birthplace in England* study.

**Results:**

Women who planned to give birth in a birth centre or at home were significantly more likely to have a normal labour and birth compared with women in the labour ward group. There were no statistically significant differences in stillbirth and early neonatal deaths between the three groups, although we had insufficient statistical power to test reliably for these differences.

**Conclusion:**

This study provides information to assist the development and evaluation of different places of birth across Australia. It is feasible to examine perinatal and maternal outcomes by planned place of birth using routinely collected linked data, although very large data sets will be required to measure rare outcomes associated with place of birth in a low risk population, especially in countries like Australia where homebirth rates are low.

## Background

There is debate in Australia and many other developed countries about the safety of different places in which to give birth. The Australian government is committed to supporting women’s choice of maternity care based on the best evidence, however there are currently no data on the comparative safety of different places of birth in this country [1]. In the United Kingdom, in an effort to provide such evidence, the *Birthplace in England* study was undertaken to compare perinatal and maternal outcomes and interventions in labour by planned place of birth at the onset of labour for women with low risk pregnancies using a composite primary outcome [2].

In Australia, most women choose hospital care for pregnancy and birth, although other options do exist, including homebirth [3-6], birth centres [7,8], stand-alone units [9], and small maternity units [10] [see definitions of place of birth]. Homebirth attracts the most debate about safety with divergent views expressed among health professionals and the community [11-13]. The main issues in Australia regarding homebirth centre around the risks to the baby, with higher perinatal mortality rates reported in some studies of homebirth [14,15] however these studies have included women with risk factors (e.g., twins, medical complications), so it is difficult to draw conclusions about low-risk women [14,16]. A study of birth centres in Australia showed that the overall rate of perinatal mortality was significantly lower in a birth centre than in a hospital irrespective of the mother’s parity however the study did not delineate between *intention* to give birth in a birth centre and actually giving birth [8].

### Definitions of place of birth

Home is where women give birth outside a formal health facility—usually in their home—and plan to receive care from a registered midwife (privately or publicly-funded). In the Birthplace in England Study, only publicly-funded homebirths were included.

A birth centre is a separate area designated to provide a home-like setting during childbirth. It is usually situated in the hospital complex. There are no private birth centres in NSW although a small number of stand-alone birth centres (n=2) do exist.

A hospital labour ward is within a hospital and is staffed by midwives and doctors. This includes public and private hospitals. In the Birthplace in England Study these were equivalent to obstetric units.

Determining the comparative safety of hospital, birth centre and home presents methodological challenges due to the self-selection of women, the timing at which they choose particular models of care and the cross-over that exists, especially between home and birth centre to hospital. In the United Kingdom it is possible to decide on place of birth at the onset of labour in pregnancies considered low risk, where a woman can decide to have her planned birth at home or she can choose to labour and birth in hospital. In Australia, the decision around place of birth is usually made prior to the onset of labour and arrangements are made accordingly. One of the significant limitations of previous studies exploring place of birth has been the lack of data on planned place of birth at the onset of labour, and about differing levels of risk and systems of care [6,17]. Many studies of homebirth and birth centre outcomes include women who chose these options early in pregnancy (around 12–16 weeks) but changed to hospital care through the pregnancy as the clinical situation changed (i.e., the development of complications). It is now recommended that women who transfer during pregnancy should be excluded from these analyses, and women should only be ‘recruited’ to studies at the onset of labour [17].

The prospective study, *Birthplace in England*, specifically examined the outcomes of women classified by place of birth at the onset of labour [2]. This study recruited more than 64,000 women with a singleton, term and booked pregnancy who gave birth between April 2008 and April 2010. Women who planned a caesarean birth and those who had caesarean births before the onset of labour, as well as women with unplanned home births were excluded. A composite primary outcome of perinatal mortality and intrapartum-related neonatal morbidities was used to compare outcomes using planned place of birth at the onset of labour (at home, freestanding midwifery units, alongside midwifery units and obstetric units). The findings showed no significant differences in the adjusted odds of the primary outcome for any of the non-obstetric unit settings compared with obstetric units, however there were differences according to parity. For women having their first baby, the odds of the primary outcome were higher for those who planned home births (adjusted odds ratio (AOR) 1.75). For multiparous women, there were no significant differences in the incidence of the primary outcome by planned place of birth. Interventions during labour were substantially lower in all non-obstetric unit settings [2].

The *Birthplace in England* study collected prospective data using a paper-based form completed by the midwife who provided intrapartum care. Additional forms were completed if an adverse outcome occurred or if the baby or mother had been admitted for higher level care. The resources required to undertake the study were considerable, including the employment of local coordinating midwives.

We were interested in determining whether we could use similar measures and analysis techniques to retrospectively examine place of birth outcomes. In many parts of Australia, there are well developed electronic perinatal data collection systems. In NSW, every birth is entered into the Perinatal Data Collection (PDC), mostly electronically at the point of care. The PDC includes all live births and stillbirths of at least 20 weeks gestation or 400g birth weight that occur in the state. It includes information on maternal demographic factors, pregnancy, labour, birth and perinatal outcomes.

Perinatal data includes only the period of the labour, birth and immediate post-partum period. Therefore access to other electronic data sets that track deaths and hospital admissions provides additional data for morbidities or deaths that occur after the early postpartum period. Data linkage enables records from different data sets to be paired [18]. This increases the scope for population-based research as routinely collected health data provides a rich source of information, and data linkage enables data from different databases, to be combined [19]. Australia does not have a unique patient (or individual) identifier, which means that common variables need to be used in the record linkage process, and these include name, date of birth, gender, address, and postcode [19,20]. Probabilistic Data Linkage assigns a weight to the result of the comparison of several variables and determines whether the records are “matched” hence belonging to the same person [19]. A code is then assigned to the linked pair, thus de-identifying the record. Mismatches can occur; where two different individuals are linked creating a false positive link, and a false negative result is where an individual’s records are not linked [19,21].

The aim of our study was firstly to determine whether a retrospective linked data study using routinely collected data was a viable means to compare perinatal and maternal outcomes and interventions in labour by planned place of birth at the onset of labour in one Australian state. To address this we aimed to undertake a similar analysis to the *Birthplace in England* study. The second aim was to report on the perinatal outcomes and labour interventions for women who planned to give birth in a hospital labour ward, birth centre or at home at the onset of labour. NSW was selected as this Australian state has the largest number of births per year, accounting for about one third of annual births in Australia [22]. From 2000-2008, the annual number of births ranged from 87,922 [23] to 96,000 [24].

## Methods

The study hypotheses were:

1) that it was possible to undertake an analysis similar to that used in the *Birthplace in England* study using a retrospective data-linkage approach

2) that for women at low risk of complications, there is no difference in perinatal mortality and morbidity associated with births planned at the onset of labour to be either at home, in a birth centre or in a standard labour ward.

The primary outcome for the second hypothesis was a composite measure that included neonatal mortality and indicators of neonatal severe morbidity. This composite outcome was the same as that used in the *Birthplace in England* study [2] and hence we did not alter it in this study.

### Study design

A secondary data analysis of routinely collected data for births from an eight-year period from mid-2000 to mid- 2008 was undertaken and, as much as possible, the analysis technique employed by the *Birthplace in England* study [2] was reproduced.

Women were eligible for inclusion in the dataset if they gave birth:

– from July 1^st^ 2000 up to and including June 30^th^ 2008;

– to a singleton baby in a cephalic presentation following spontaneous labour at > 37 weeks gestation.

Women were excluded if:

– they had an elective caesarean section;

– the baby was born before arrival to hospital;

– the birth occurred before 37 completed weeks gestation;

– they had received no antenatal care;

– they were attempting a vaginal birth after previous caesarean section (VBAC) for this birth;

– the baby was diagnosed with a congenital abnormality (that is, registered on the NSW *Register of Congenital Conditions*);

– they had their labour induced for any reason.

– Any baby who had received a diagnosis (as recorded on the birth and subsequent admission data on the Admitted Patient Data Collection) of a congenital condition and who died within the first week of life resulted in that woman and baby pair being excluded from the cohort.

### Ethical approval

Ethical approval was obtained from the NSW Population and Health Services Research Ethics Committee, Protocol No. 2010/12/291.

### Data sources

There were five data collections used in the linkage. The data from each collection will be further described later in the paper.

1) *NSW Perinatal Data Collection* (PDC). The PDC is a population-based surveillance system containing maternal and infant data on all births of greater than 400 g birth weight or 20 weeks gestation and covers over one-third of births which occur in Australia^1^. Perinatal data recorded in the MDC during the study data collection were provided by NSW Department of Health.

2) *NSW Admitted Patient Data Collection* (APDC). The APDC records all admitted patient services provided by NSW Public Hospitals, Public Psychiatric Hospitals, Public Multi-Purpose Services, Private Hospitals, and Private Day Procedure Centres. The clinical data component of the APDC utilises the International Classification of Diseases – Australian modification (ICD-10-AM). Data on all hospital admissions were provided by the APDC.

3) *NSW Register of Congenital Conditions* (*RCC*). This register collects data on all diagnosed congenital abnormalities. Data regarding congenital abnormalities were available for all births occurring in the study period.

4) NSW Registry of Births, Deaths and Marriages (NSWRBDM) provides data on all registered birth and deaths.

5) The Australian Bureau of Statistics (ABS) provides data on deaths including primary cause and date of death.

### Data linkage method

Probabilistic linkage of the five datasets was undertaken by the Centre for Health Record Linkage. Probabilistic record linkage software works by assigning a ‘linkage weight’ to pairs of records [25]. For example, records that match perfectly or nearly perfectly on first name, surname, date of birth and address have a high linkage weight, and records that match only on date of birth have a low linkage weight. If the linkage weight is high it is likely that the records truly match, and if the linkage weight is low it is likely that the records are not a match. This technique has been shown to have a false positive rate of 0.3% of records [25]. The collections were linked to ensure that deaths or significant morbidity requiring hospitalisation occurring post-perinatal data collection could be included.

### Data definitions and collections

Women were classified according to planned place of birth as recorded on the PDC. Place of birth was defined as hospital labour ward, birth centre or home [see definitions of place of birth]. The exclusion criteria used enabled this planned place of birth to approximate the planned place of birth at the onset of labour as closely as a retrospective dataset can allow, with all women in this cohort labouring spontaneously at ≥37 weeks gestation.

The primary composite outcome was defined as an infant having any one of the following [2]:

– Stillbirth

– Early neonatal death (<7 days)

– Neonatal encephalopathy

– Meconium aspiration syndrome

– Brachial plexus injury

– Fractured clavicle

– Fractured humerus

Stillbirths or neonatal deaths (NND) were identified in the PDC, APDC, RBDM and ABS. Only NNDs that occurred within the first week following birth were included in line with the *Birthplace in England* study methodology. To determine the remaining components of the composite outcome the following ICD-10-AM codes from the birth as well as any subsequent admission within the time frame were searched in the APDC: neonatal encephalopathy (P91.6. P91.81), meconium aspiration syndrome (P24.0 and P24.9), brachial plexus injury (P14.0, P14.1, P14.2 and P14.3), fractured humerus (P13.3), and fractured clavicle (P13.4). The primary outcome will be reported as incidence per 1000 births.

### Data analysis

Women were classified into the three groups according to their planned place of birth at the onset of labour and the primary composite outcome for the neonate was used [2]. An additional analysis separating multiparous and primiparous women was undertaken as well as an analysis of stillbirth and early neonatal death. Data were analysed for all women and then for women without complications at the start of labour.

Continuous data were summarised using means derived from t-tests or ANOVA. Chi-square analyses were conducted on contingency data. Logistic regression was undertaken with adjustments occurring for maternal age, gestational age in weeks at delivery and parity as in the *Birthplace in England* study [2]. For all analyses, women in the labour ward group were the reference category. Cases with missing data were removed from the rates and regression calculations.

Data were analysed with IBM SPSSv.20® with statistical significance established at the p < 0.005 level. The cohort of women identified as complicated were those who had pre-existing or pregnancy related hypertension or diabetes as recorded on the PDC or had either an antenatal admission or a birth admission (as recorded on the APDC) which included the ICD-10-AM codes O14.0, O14.1, O14.2, O14.9 (proteinuric hypertension), O15.0 (antenatal eclampsia), O10.0, O10.1, O10.2, O10.3, O10.4, O11 or O10.9 (chronic hypertension), O13.0 (gestational hypertension), O24.0, O24.1, O24.2, O24.3 (Type I or Type II diabetes) or O24.4, O24.9 (gestational diabetes). Women who had prolonged rupture of membranes were identified from the APDC utilising the codes O42.1 and O42.2 as were women who had experienced an antepartum haemorrhage (O46.0, O46.8 or O46.9). These were the same measures of morbidity as in *Birthplace in England* study [2].

To ensure accuracy of pregnancy classification, the birth and subsequent admissions of all women and babies which resulted in either a stillbirth or a NND were searched per data line. If a pregnancy complication or a pre-existing complication (other than those already listed) was noted on any woman’s record her case was coded as complicated if it was not already so.

## Results

In total, 258,161 women and neonates were included in the analysis. The majority of women (94.1%; 242,936) had their planned place of birth recorded as in a hospital labour ward, with 5.6% (14, 483) in a birth centre and 0.3% (n = 742), planning a homebirth. There were differences in the demographic characteristics of the women in the three groups. Women who planned homebirth were more likely to be older (mean 32 years; standard deviation (SD) = 5.23) than those in the labour ward group (mean 29 years; SD = 5.67) or those in the birth centre (mean 30 years; SD = 5.29) and less likely to be primiparous (42% versus 63% and 62%) (p < 0.001) (Table 1). More women in the homebirth and birth centre groups had a gestational age of 42 weeks or more compared with the labour ward group (8.4% and 2.9%; 1.4%) (p < 0.001).

**Table 1:** Demographic characteristics and complications prior to labour by planned place of birth

	**Hospital n = 242 936**	**Birth centre n = 14 483**	**Home n = 742**	**P**
Maternal age (years)				
Mean (SD) #	29.2 (5.67)	30.0 (5.29)	32.4 (5.23)	<0.001
<20	15 280 (6.3%)	513 (3.5%)	7 (0.9%)	
20-24	42 544 (17.5%)	2 122 (14.7%)	54 (7.3%)	
25-29	73 440 (30.2%)	4 529 (31.3%)	159 (21.4%)	<0.001
30-34	73 404 (30.2%)	4 788 (33.1%)	249 (33.6%)	
35-39	32 058 (13.2%)	2 181 (15.1%)	185 (24.9%)	
≥40	6 134 (2.5%)	349 (2.4%)	49 (6.6%)	
Missing	76 (0.03%)	1 (0.09%)	39 (5.3%)	
Previous pregnancies (≥20 weeks)				
0	149 459 (61.5%)	9 145 (63.1%)	313 (42.2%)	
1	54 445 (22.4%)	3 328 (23.0%)	219 (29.5%)	<0.001
2	24 627 (10.1%)	1 453 (10.0%)	143 (19.3%)	
≥3	14 259 (5.9%)	552 (3.8%)	59 (8.0)	
Missing	146 (0.06%)	5 (0.04%)	8 (1.1%)	
Gestation (completed weeks)				
Mean (SD) #	39.5 (1.10)	39.8 (1.12)	39.9 (1.16)	<0.001
37	12441 (5.1%)	541 (3.7%)	22 (3.0%)	<0.001
38	31268 (12.9%)	1424 (9.8%)	55 (7.4%)	
39	62137 (25.6%)	3405 (23.5%)	147 (19.8%)	
40	95992 (39.5%)	5353 (37.0%)	309 (41.6%)	
41	37682 (15.5%)	3347 (23.1%)	147 (19.8%)	
≥42	3416 (1.4%)	413 (2.9%)	62 (8.4%)	
Complication conditions prior to labour				
Prolonged rupture of membranes*	72 (0.03%)	0	0	0.11
Preeclampsia	2579 (1.1%)	93 (0.6%)	1 (0.1%)	<0.001
Gestational hypertension	6997 (2.9%)	322 (2.2%)	1 (0.1%)	<0.001
Chronic hypertension	685 (0.3%)	29 (0.2%)	2 (0.3%)	0.38
Antepartum haemorrhage	1293 (0.5%)	64 (0.4%)	3 (0.4%)	0.49
Eclampsia	11 (<0.01%)	0	0	0.85
Gestational diabetes**	32 (0.01%)	0	2 (0.3%)	0.36
Pre-pregnancy diabetes (Type I)	149 (0.06%)	0	0	0.03
Complications per woman				
0	221284 (91.1%)	13725 (94.8%)	735 (99.1%)	
1	18460 (7.6%)	637 (4.4%)	6 (0.8%)	<0.001
≥2	3192 (1.3%)	121 (0.8%)	1 (0.1%)	

#Utilising ANOVA.

*Prolonged rupture of membranes: >24 hours.

**Any form of GDM.

Overall, the incidence of the primary outcome was not statistically different between the groups (Table 2). Nonetheless, the incidence of the primary outcome for nulliparous women in the homebirth group was higher than for other groups, as was seen in the *Birthplace in England* study. There were four babies with adverse outcomes identified in this group (two of these were >42 weeks and one >41 weeks). Three of the four babies were born in hospital. These babies were admitted to a Neonatal Intensive Care Unit or Special Care Nursery for between two and eleven days. Following this, they were all discharged alive and not readmitted. Babies of multiparous women were significantly less likely to experience the primary outcome in the birth centre setting (AOR 0.45; 95% CI 0.26-0.81) compared with in the labour ward. For multiparous women, the incidence of the primary outcome in the homebirth group was similar to the birth centre group (Table 2).

**Table 2:** Primary composite outcome by planned place of birth at the onset of labour

**Planned place of birth**	**No. events/births**	**Incidence of events/1000**	**Unadjusted OR**	Adjusted OR*
**All women**				
Total*	1 481/258 045	5.7		
Hospital	1 399/242 860	5.8	1.00	1.00
Birth centre	77/14 482	5.3	0.92 (0.73-1.16)	0.87 (0.69-1.10)
Home Birth	5/703	7.1	1.17 (0.49-2.83)	1.06 (0.44-2.56)
Nulliparous women	1 018/158 866	6.4		
Hospital	949/149 417	6.4	1.00	1.00
Birth Centre	65/9 145	7.1	1.12 (0.87-1.44)	1.04 (0.81-1.34)
Home Birth	4/304	13.2	2.03 (0.75-5.44)	1.72 (0.64-4.63)
Multiparous women	461/99 022	4.7		
Hospital	448/93 298	4.8	1.00	1.00
Birth Centre	12/5 332	2.3	0.47 (0.26-0.83)	0.45 (0.26-0.81)
Home Birth	1/392	2.6	0.49 (0.07-3.52)	0.47 (0.07-3.38)
**Women without complications at start of care in labour**				
Total*	1291/235 611	5.5		
Hospital	1221/221 193	5.5	1.00	1.00
Birth centre	66/13 723	4.8	0.87 (0.68-1.12)	0.82 (0.64-1.05)
Home birth	4/695	5.8	0.99 (0.37-2.64)	0.87 (0.33-2.35)
Nulliparous women	887/144 830	6.1		
Hospital	829/135 897	6.1	1.00	1.00
Birth centre	55/8 633	6.4	1.05 (0.80-1.37)	0.96 (0.73-1.26)
Home birth	3/300	10.0	1.60 (0.51-4.99)	1.31 (0.42-4.11)
Multiparous women	403/90 632	4.5		
Hospital	391/85 159	4.6	1.00	1.00
Birth centre	11/5 085	2.2	0.47 (0.26-0.86)	0.46 (0.25-0.83)
Home birth	1/388	2.6	0.52 (0.07-3.72)	0.50 (0.07-3.60)

*Logistic regression was undertaken with adjustments occurring for maternal age, gestational age in weeks at delivery and parity. Any case with missing data was excluded from the regression.

The next analysis used the same composite primary outcome but did not include women who had a complication at the onset of labour. Overall, there were no differences in the primary outcome. However, in multiparous women, the birth centre had a protective effect compared with the labour ward group (AOR 0.46; 95% CI 0.25-0.83), but with no statistically significant difference for nulliparous women (AOR 0.96; 95% CI 0.73-1.26) (Table 2).

While the *Birthplace in England* study examined only the composite primary outcome, we also examined the stillbirth and early neonatal death rates. There were no statistically significant differences between the three groups, nor any differences for nulliparous or multiparous women (Table 3). Once women with complications were excluded, the stillbirth and early neonatal death rates were still not significantly different between the groups. There were no stillbirths or neonatal deaths in the homebirth group for women without complications.

**Table 3:** Stillbirth during labour and early neonatal death by planned place of birth

**Planned place of birth**	**No. events/births**	**Incidence of events/1000**	**Unadjusted OR**	**Adjusted OR**
**All women**				
Total*	266/257 834	1.03		
Hospital	255/242 665	1.05	1.00	1.00
Birth centre	10/14 476	0.69	0.66 (0.35-1.24)	0.66 (0.35-1.24)
Home birth	1/693	1.44	1.28 (0.18-9.17)	1.29 (0.18-9.23)
Nulliparous women	169/158 866	1.06		
Hospital	158/149 417	1.06	1.00	1.00
Birth centre	10/9 145	1.09	1.03 (0.55-1.96)	0.99 (0.52-1.88)
Home birth	1/304	3.29	3.03 (0.42-21.70)	2.48 (0.34-18.02)
Multiparous women	96/99 022	N/A	N/A	N/A
Hospital	96/93 298			
Birth centre	0/5 332			
Home birth	0/392			
**Women without complications at start of care in labour**				
Total*	206/235 462	0.87		
Hospital	198/221 056	0.90	1.00	1.00
Birth centre	8/13 718	0.58	0.65 (0.32-1.32)	0.63 (0.31-1.28)
Home birth	0/688	0.00		
Nulliparous women	129/144 830	0.89		
Hospital	121/135 897	0.89	1.00	1.00
Birth centre	8/8 633	0.93	1.04 (0.51-2.13)	0.95 (0.46-1.96)
Home birth	0/300	0.00		
Multiparous women	77/90 632	N/A	N/A	N/A
Hospital	77/85 159			
Birth centre	0/5 085			
Home birth	0/388			

*Cases with missing data were not included in rates or regression calculations.

N/A due to being unable to calculate a statistic due to zero events in the Birth Centre and Home Birth groups.

Just over 29% of women were transferred from the birth centre and almost one fifth (18.6%) transferred from home to a labour ward or operating theatre (Table 4). Nulliparous women were more likely to be transferred compared with multiparous women. These rates and differences related to parity were similar for women without complications at the onset of labour.

**Table 4:** Proportion of women transferred from home or birth centres to labour wards

	**Birth centre**	**Home**
**All women**		
All women	4 322/14 483 (29.8%)	138/742 (18.6%)
Nulliparous women	3 551/9 145 (38.8%)	101/313 (32.3%)
Multiparous women	770/5 333 (14.4%)	37/427 (8.7%)
**Women without complications**		
All women	3 980/13 724 (29.0%)	135/735 (18.4%)
Nulliparous women	3 267/8 633 (37.8%)	99/309 (32.0%)
Multiparous women	712/5 086 (14.0%)	36/424 (8.5%)

Women in the homebirth and birth centre groups were significantly more likely to have a spontaneous vaginal birth (97% vs 86%) compared to the labour ward group (74%) and less likely to require an instrumental vaginal birth (4% and 9% compared with 16%) or an intrapartum caesarean section (3% and 5% compared with 10%) (Table 5). The rates of intervention during labour and birth were significantly lower for women in the birth centre or homebirth groups on all outcomes except severe perineal trauma, where there was no statistical difference (Table 5). In particular, the rates of syntocinon augmentation, epidural or spinal analgesia for labour, and episiotomy were all significantly lower in the birth centre and homebirth groups compared with the labour ward group.

**Table 5:** Type of birth and intervention rates by planned place of birth- all women*

**Intervention and planned place of birth**	**No. events/births**	**Incidence of events/100 births**	**Unadjusted**	**Adjusted**
Spontaneous vertex birth	192 432/257 888	74.6		
Hospital	179 307/242 715	73.9	1.00	1.00
Birth centre	12 447/14 477	86.0	2.17 (2.07-2.28)	2.73 (2.60-2.87)
Home birth	678/696	97.4	3.76 (2.91-4.86)	4.05 (3.09-5.32)
Ventouse delivery	25 940/257 888	10.1		
Hospital	25 060/242 715	10.3	1.00	1.00
Birth centre	867/14 477	6.0	0.55 (0.52-0.60)	0.51 (0.47-0.54)
Home birth	13/696	1.9	0.16 (0.09-0.27)	0.18 (0.10-0.30)
Forceps delivery	13 188/257 888	5.1		
Hospital	12 705/242 715	5.2	1.00	1.00
Birth centre	468/14 477	3.2	0.61 (0.55-0.66)	0.55 (0.50-0.60)
Home birth	15/696	2.2	0.37 (0.22-0.62)	0.44 (0.26-0.73)
Intrapartum caesarean section	26 385/257 888	10.2		
Hospital	25 669/242 715	10.6	1.00	1.00
Birth centre	693/14 477	4.8	0.43 (0.39-0.46)	0.36 (0.34-0.39)
Home birth	23/696	3.3	0.27 (0.18-0.41)	0.27 (0.17-0.40)
3rd or 4th degree perineal trauma**				
with episiotomy extensions	7 557/231 403	3.3		
Hospital	7 083/216 955	3.3	1.00	1.00
Birth centre	461/13 782	3.3	1.03 (0.93-1.13)	0.93 (0.84-1.02)
Home birth	13/666	2.0	0.55 (0.32-0.96)	0.66 (0.38-1.14)
Syntocinon augmentation	51 719/257 888	20.1		
Hospital	50 067/242 715	20.6	1.00	1.00
Birth centre	1 612/14 477	11.1	0.48 (0.46-0.51)	0.43 (0.41-0.45)
Home birth	40/696	5.7	0.22 (0.16-0.30)	0.24 (0.17-0.33)
Epidural or spinal analgesia for labour	73 012/257 888	28.3		
Hospital	70 635/242 715	29.1	1.00	1.00
Birth centre	2 321/14 477	16.0	0.47 (0.44-0.49)	0.40 (0.37-0.41)
Home birth	56/696	8.1	0.20 (0.15-0.26)	0.19 (0.15-0.26)
General anaesthesia ***	4 429/26 365	16.8		
Hospital	4 357/25 649	17.0	1.00	1.00
Birth centre	70/693	10.1	0.55 (0.43-0.71)	0.61 (0.48-0.79)
Home birth	2/23	8.7	0.47 (0.11-1.99)	0.71 (0.16-3.06)
Episiotomy **	41 885/231 396	18.1		
Hospital	40 506/216 948	18.7	1.00	1.00
Birth centre	1 353/13 782	9.8	0.47 (0.45-0.50)	0.41 (0.39-0.44)
Home birth	26/666	3.9	0.17 (0.11-0.25)	0.18 (0.12-0.26)

*Cases with missing data were not included in rates or regression calculations.

**Denominator = vaginal birth.

***Denominator = caesarean section.

The final analysis examined the incidence of normal labour and birth by planned place of birth. We have defined ‘normal labour and birth’ as spontaneous onset of labour, no epidural or spinal analgesia, normal vaginal birth (no forceps or vacuum extraction or caesarean section) and no episiotomy. Women who planned to give birth in a birth centre or at home were significantly more likely to have a normal labour and birth compared with women in the labour ward group (Table 6). This was maintained once women with known complications were excluded.

**Table 6:** Incidence of normal labour and birth by planned place of birth

**Planned place of birth**	**No. events/births**	**Incidence of events/1000**	**Unadjusted OR**	**Adjusted OR**
**All women**				
Total	117 447/257 888	45.5		
Hospital	106 869/242 715	44.0	1.00	1.00
Birth centre	9 948/14 377	69.2	2.79 (2.69-2.90)	3.51 (3.38-3.65)
Home birth	630/696	90.9	7.16 (5.86-8.76)	8.00 (6.46¬-9.90)
**Women without complications at start of care in labour**				
Total	108 167/235 462	45.9		
Hospital	98 050/221 056 9	44.4	1.00	1.00
Birth centre	495/13 718	69.2	2.82 (2.72-2.93)	3.54 (3.41-3.69)
Home birth	622/688	90.4	6.98 (5.71-8.54)	7.82 (6.31-¬9.69)

NB: Normal labour and birth – spontaneous labour, no epidural or spinal, general anaesthesia, forceps, vacuum extraction or episiotomy for the mother.

## Discussion

The aim of our study was to determine firstly, whether a retrospective linked data study was a viable alternative to such a design using routinely collected data in one Australian state and secondly, to report on the outcomes and interventions for women (and their babies) who planned to give birth in a hospital labour ward, birth centre or at home.

### Limitations of sample size

This study was exploratory in nature – we undertook this analysis on data from the most populous state in Australia to establish that the approach was feasible before expanding it to the whole country. Therefore, we did not undertake sample size calculations and recognise that the study is underpowered to draw conclusions about homebirth and rare perinatal outcomes. This study aimed to determine whether a retrospective linked data study using routinely collected data was a viable means to compare perinatal and maternal outcomes and interventions in labour by planned place of birth in one Australian state using the composite primary outcomes outlined in the *Birthplace in England Study*. In order to fulfill this endeavour, we were limited to the outcomes from the study.

It is important to note that in all instances where we mentioned a lack of statistical significance in comparisons between the three groups we had ≤40% statistical power to detect any true differences. The small number of women choosing to give birth at home in this analysis reflects the Australian context. For example, in the most recent data from NSW, 95.5% (n = 90,730) of women planned to give birth in a hospital labour ward, 3.7% (n = 3,533) planned to give birth in a birth centre and only 0.3% (n = 264) planned to give birth at home [26].

*Birthplace in England*[2] calculated a sample size a priori based on the incidence of the composite measure of any neonatal morbidity, the primary outcome for the study, as being 3.6 per 1,000 births. They calculated that in order to have adequate power to detect important clinical differences in outcome that are associated with planned place of birth, they would need to collect data on at least 20,000 ‘low risk’ women planning to give birth in an obstetric unit, at least 17,000 women planning to give birth at home and at least 5,000 women planning to give birth in each type of midwifery unit [27]. Given these figures, it would take more than 30 years of data collection in NSW to have an adequate sample size. Despite the sample size limitation, this remains the largest comparison of place of birth including homebirth in Australia.

### Using routinely collected data to study planned place of birth outcomes

The study has demonstrated that, despite the sample size limitations, it is feasible to undertake a planned place of birth study using routinely collected linked data. There are considerable resource implications of using routinely collected data especially as such analyses can be repeated relatively quickly at different time points to track trends. Using linked data has added advantages. Linking datasets improves accuracy as well as provides a more comprehensive picture of the medical history and any events after the birth [28].

There are disadvantages of using routinely collected linked data compared with specifically collected prospective data. While population health databases are an easily accessible and available resource, they were not designed to answer a specific research question, especially one applied retrospectively [28]. There are also questions about the quality of the data. In NSW over the past decade there have been nine published reports assessing the validity of the NSW Perinatal Data Collection [29]. A systematic review of the quality of data in perinatal population health databases found that, in general, events related to labour and birth were accurately reported and the level of ascertainment increases as time to birth decreased [29]. In NSW, the accuracy of neonatal morbidity and mortality in routinely collected datasets such as used in our study have been reported to be higher than maternal conditions [30,31].

This analysis could only use data which was included in the datasets and hence could be linked. It is possible that there were unreported homebirths in NSW during this time period and these could not therefore be linked. Nonetheless, it is likely that these numbers are very small and were more likely not to have been attended by midwives (freebirths).

We used a similar composite neonatal outcome to the *Birthplace in England* study. Composite indicators in population health datasets are becoming more common. In one recent study from the same state, NSW, a Neonatal Adverse Outcome Indicator (NAOI) was found to be able to reliability identify infants with a severe neonatal outcome and could be a useful way to monitor trends in maternity care in a cost effective manner [32].

A further issue is that the planned place of birth variable as recorded in the PDC cannot be guaranteed to equate to the planned birth of birth at labour onset. Women who choose to give birth at home or in birth centres are on the whole strongly committed to this birth choice and where there are no complications, as is seen in this cohort, women are unlikely to change their minds after 37 weeks. Taking a term gestational age (37weeks) as intended place of birth also reduces the problems seen in previous studies with choice of birth place documented at booking [14]. The outcomes of this study approximate the *Birthplace in England* study which is reassuring but validation work on the accuracy of intended place of birth and onset of labour would be valuable. Actual place of birth is accurate as it is a retrospective outcome.

While it was the intention of the *Birthplace in England* study not to include inductions of labour it was not always possible to determine whether induction of labour, previous caesarean section and known group B strep carriage could be identified or reliably inferred from the data for 2008. The data collection form for the study was modified in 2009 to capture data on induction of labour and previous caesarean section [2]. As induction of labour means an added layer of risk we have excluded all these woman in this study. In addition, inductions of labour do not occur at home, and usually not in a birth centre, therefore it was the more conservative option.

### Neonatal and maternal outcomes by planned place of birth

The second aim of the study was to determine the outcomes for term babies according to planned place of birth. In Australia, homebirth is a much less common choice than in many other countries due to a combination of access, cultural history and professional support [33]. This means that the overall numbers of women planning to give birth at home is insufficient to be able to make definitive conclusions about the safety of homebirth. The *Birthplace in England* study recruited 16,840 women planning to give birth at home at the onset of labour. Even in an 8-year time period, we were only able to include 742 women planning to give birth at home at the same time point. Nonetheless, the study provides the largest analysis of planned homebirth and birth centre births at the onset of labour in Australia and as such provides valuable evidence to assist the development of services and to support the need for ongoing data collection and research.

Our analysis, despite the sample size limitations, suggests that women planning to give birth at home, hospital or in a birth centre can expect to have good outcomes. The analysis by parity indicated that there were no statistically significant differences in adverse neonatal outcomes for nulliparous women although the numbers are much smaller than the *Birthplace in England* study. The neonatal outcomes for women giving birth centre or a labour ward were comparable although the levels of intervention were higher in the labour ward groups despite similarities in demographic and obstetric predictors.

Multiparous women had good outcomes regardless of planned place of birth. However, there was significantly less intervention for this group with no apparent differences in neonatal outcomes suggesting that it is reasonable to support home as a place of birth for multiparous women. Similar findings were found in the *Birthplace in England* study where multiparous women who chose to give birth at home had favourable outcomes and less perinatal interventions [2].

Another issue raised by following the *Birthplace in England Study* methodology is the much higher incidence of women >42 weeks in the homebirth group. Two out of the four adverse events in the homebirth group were associated with women who were > 42 weeks and one was >41 weeks. One could argue pregnancy beyond 42 weeks is not low risk and hence should not be included in the low risk group. Women who choose to give birth at home and in birth centres tend to want a more natural approach to labour and birth and are more likely to avoid intervention. The question that needs to be asked is whether this a reflection on the safety of place of birth or the different choices women make in these models of care. It would be worthwhile keeping this in mind in future studies.

Transfers during labour from home or birth centre to hospital require further analysis. Further research needs to be undertaken to determine why the high transfer rates were seen and to what extent these contribute to the higher, non-significant rates, for nulliparous women. It would be important to understand whether these rates are related to inadequate counseling or inadequate screening or a combination of factors.

This analysis cannot distinguish between type of provider. For example, it is not possible to determine whether the homebirths were part of a publicly-funded homebirth program or attended by privately practising midwives. There are currently four publicly-funded homebirth programs in NSW although most were established post 2006 which means they would not have contributed large numbers of women to this cohort [34]. Privately practising midwives have not had access to professional indemnity insurance since 2001 which means the number of women accessing homebirth through this provider group has remained low. These factors have contributed to the low numbers of women choosing to give birth at home.

## Conclusion

This study has demonstrated that it is possible to study the outcomes of planned place of birth using routinely collected linked data. Despite its limitation in sample size, especially for homebirth and rarer outcomes, the study provides important information that could inform the development, data collection processes and evaluation of different places of birth across Australia. Very large data sets will be required to measure rare outcomes associated with place of birth in the low risk population, especially in countries like Australia where homebirth rates are low.

### Details of ethics approval

Ethical approval was obtained from the NSW Population and Health Services Research Ethics Committee, Protocol No. 2010/12/291.

## Abbreviations

ABS: Australian Bureau of Statistics; APDC: Admitted patient data collection; CHeReL: Centre for health record linkage; NICU: Neonatal Intensive Care Unit; NND: Neonatal death; NSW: New South Wales; PDC: Perinatal data collection; RBDM: Registry of births, deaths and marriages; RCC: Register of congenital conditions; SCN: Special care nursery; VBAC: Vaginal birth after caesarean.

## Competing interests

The authors declare that they have no competing interests.

## Authors’ contributions

The Birthplace in New South Wales Pilot Study was formulated by The Birthplace in Australia Chief Investigator Committee which includes all authors. CH and HD conceived of the study design, and wrote the first draft of the paper. HD and CT acquired the data and undertook the analysis and contributed to the content and editing. DS provided post-hoc calculations and contributed to the content of the paper. DE, JO, MF, HM, DF and VS contributed to the content and editing of the paper. All authors agree with the final draft and accept responsibility for the content of the paper.

## Pre-publication history

The pre-publication history for this paper can be accessed here:

http://www.biomedcentral.com/1471-2393/14/206/prepub
